# Supplementary material for: Identification of photoperiod-induced specific miRNAs in the adrenal glands of Sunite sheep (Ovis aries)
Source: Front Vet Sci. 2022 Jul 22;9:888207. doi: 10.3389/fvets.2022.888207 (PMC9354845; doi:10.3389/fvets.2022.888207)
Supplement: Supplementary file 1 [file Data_Sheet_1.ZIP › Supplementary file/miRDeep2 file.zip!/miRDeep2 file/Novel_Structure/LP42A1/Novel_506.pdf]

Provisional ID	:	NC_019477.2_29137
Score total	:	1.6
Score for star read(s)	:	-1.3
Score for read counts	:	0
Score for mfe	:	1.3
Score for randfold	:	1.6
Score for cons. seed	:	
Total read count	:	21
Mature read count	:	20
Loop read count	:	0
Star read count	:	1

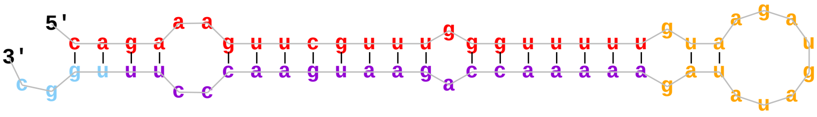

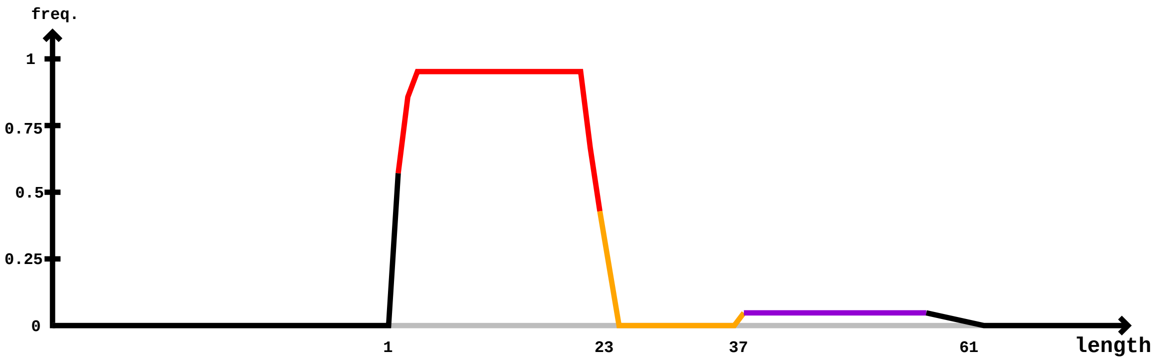


```
                                    Mature                              Star
5'- uaagaaaauaaaagacuaaaaauauuggauuggccagaaaguucguuuggguuuuuguaagaugauauagaaaaaccagaaugaacccuuuggccaacuuaauauaauac   -3'    obs
    uaagaaaauaaaagacuaaaaauauuggauuggccagaaaguucguuuggguuuuuguaagaugauauagaaaaaccagaaugaacccuuuggccaacuuaauauaauac          exp
    .....................(((((((.(((((((((..((((((((.(((((((.((........)).))))))).))))))))..))))))))).))))))).....   reads   mm   sample
    ..................................caAaaaguucguuuggguuuu.......................................................   5       1    seq
    ..................................caAaaaguucguuuggguuuuu......................................................   7       1    seq
    ...................................aAaaaguucguuuggguuu........................................................   6       1    seq
    ....................................gaaaguucguuuggguuuuu......................................................   1       0    seq
    ....................................gaaaguucguuuggguuuuuC.....................................................   1       1    seq
    ......................................................................aaaaaccagaaugaacccuu....................   1       0    seq
```
